# Telehealth with Older Adults Curriculum Development and Implementation

**DOI:** 10.1093/geroni/igaf122.1635

**Published:** 2025-12-31

**Authors:** Joan Ilardo, Angela Zell

**Affiliations:** Michigan State University, East Lansing, Michigan, United States; Michigan State University, East Lansing, Michigan, United States

## Abstract

The SEMCOAV Telehealth curriculum was developed based on insights gathered from focus groups with older adults in both urban and rural areas. These groups provided valuable input on what doctors should know to conduct telehealth visits that effectively meet the needs of older patients. Their feedback directly shaped the curriculum to ensure it addressed real-life needs of older adults. Content aligns with the six telehealth competencies outlined by the Association of American Medical Colleges. We designed six digitally recorded modules: 1) Setting Expectations, 2) Patient Needs and Preferences, 3) Communication via Telehealth, 4) Techniques for Evaluating Older Patients During a Telehealth Visit, 5) Technology for Telehealth, and 6) Ethics and Legal Considerations of Telehealth. Each module is paired with a facilitator guide, ensuring ease of use and effectiveness in teaching. Residents from four family and internal medicine programs completed pre- and post-surveys to assess their knowledge before and after viewing the modules. Feedback was overwhelmingly positive. Residents reported the modules were helpful, and residency program directors expressed satisfaction with the added value these modules brought to their curriculum. The modules provided both new information and reinforced residents’ clinical experiences conducting telehealth visits with older adult patients. Our project has the potential to improve access to quality, convenient care, reduce no-show rates, lower readmissions, minimize unnecessary emergency visits, and enhance care plan adherence, ultimately leading to improved clinical outcomes, higher patient and provider satisfaction, and reduced patient costs, such as transportation and time off work.

